# Mammographycally occult high grade ductal carcinoma *in situ* (DCIS) as second primary breast cancer, detected with MRI: a case report

**DOI:** 10.2478/v10019-010-0033-9

**Published:** 2010-08-09

**Authors:** Marta Zebic-Sinkovec, Maksimiljan Kadivec, Gasper Podobnik, Erik Skof, Marko Snoj

**Affiliations:** 1 Department of Radiology, Institute of Oncology Ljubljana, Slovenia; 2 Department of Medical Oncology, Institute of Oncology Ljubljana, Slovenia; 3 Department of Surgery, Institute of Oncology Ljubljana, Slovenia

**Keywords:** high-grade DCIS, second primary breast cancer, MRI

## Abstract

**Background:**

Contralateral breast cancer (CLB) is the most common second primary breast cancer in patients diagnosed with breast cancer. The majority of patients harbouring CLB tumours develop the invasive disease. Almost all invasive carcinomas are believed to begin as ductal carcinoma in situ (DCIS) lesions. The sensitivity of MRI for DCIS is much higher than that of mammography.

**Case report:**

We report the case of a woman who was treated with breast conserving therapy 10 years ago. At that time the invasive medullary carcinoma was diagnosed in the left breast. Ten years later mammographically occult DCIS was diagnosed with MRI-guided core biopsy in contralateral breast.

**Conclusions:**

There might be a potential role of MRI screening as part of an annual follow-up for patients diagnosed with breast cancer.

## Introduction

Contralateral breast cancer (CLB) is the most common second primary breast cancer in patients diagnosed with breast cancer.^1^ The annual risk of developing any CLB remains constant at approximately 0.75% and persists for at least 20 years after the treatment. The majority of patients (83%) harbouring CLB tumours develop the invasive disease.^2^ There is little data on the use of MRI as a screening tool to detect a recurrence after the breast-conserving therapy. Gorechland *et al.* concluded that MRI screening would not have been cost-effective and was unlikely to have improved the overall survival.^3^ However, the role of MRI in detection of invasive carcinoma had already been known, Kuhl *et al.* published in 2007 that MRI is more sensitive for detecting ductal carcinoma *in situ* (DCIS) than mammography (92% vs. 56%), especially for high-grade DCIS without necrosis (92% vs. 35%).^4^ Almost all invasive carcinomas are believed to begin as DCIS lesions.^5^ Therefore, some invasive carcinomas can be prevented by a timely intervention on the basis of MRI findings.

## Case report

**FIGURE 1.**
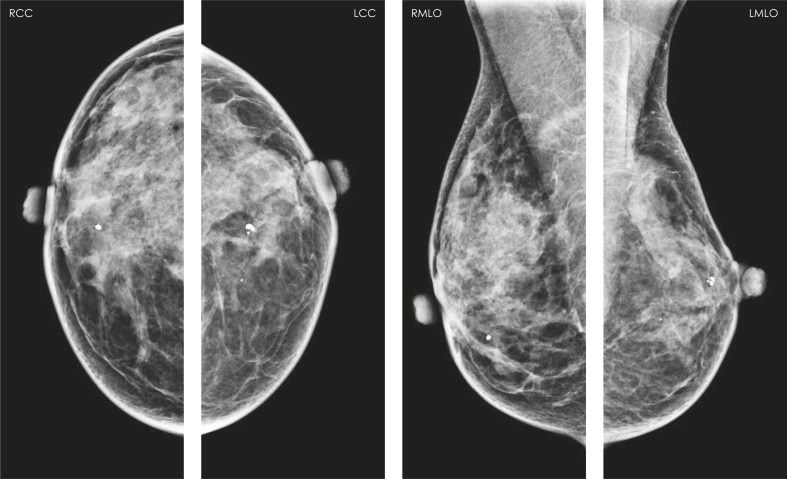
The mammograms were categorized as BI-RADS 2 (cyst, benign calcifications, postoperative changes). The breast density was categorized as ACR III. There was no change in comparison with previous mammograms.

A 47-year-old female patient was treated by breast conserving surgery in 1999. At that time invasive medullary carcinoma was diagnosed in her left breast. The dissection of axilla has been done and there was no metastatic lymph node. She received adjuvant chemotherapy and a radiation therapy. She had the regular clinical and mammographic follow-up. In April 2009 her last mammography was obtained (Figure 1). Radiological findings were evaluated according to the Breast Imaging Reporting and Data System by American College of Radiology (Figure 1).

In November 2009, she visited her doctor earlier because of changes in her right nipple. The nipple became retracted. She also had pain in her breast. Breast MRI was performed, using a 1.5-T magnet with a dedicated bilateral breast surface coil with prone position. The imaging protocol and parameters were as follows: axial T1-weighted image and short-tau inversion recovery (STIR) of both breasts were obtained with 3 mm slice thickness. Next, T1-weighted images were acquired using a 3D fast low angle shot (FLASH) through both breasts. Pre-contrast images were obtained in the axial plane with a slice thickness of 1.0 mm with a distance factor 20% before the administration of the contrast agent. Then, five sequential contrast-enhanced images were acquired at every 1 min 23 s. The MRI findings were categorized according to Breast Imaging Reporting and Data system (BI-RADS) lexicon.

**FIGURE 2.**
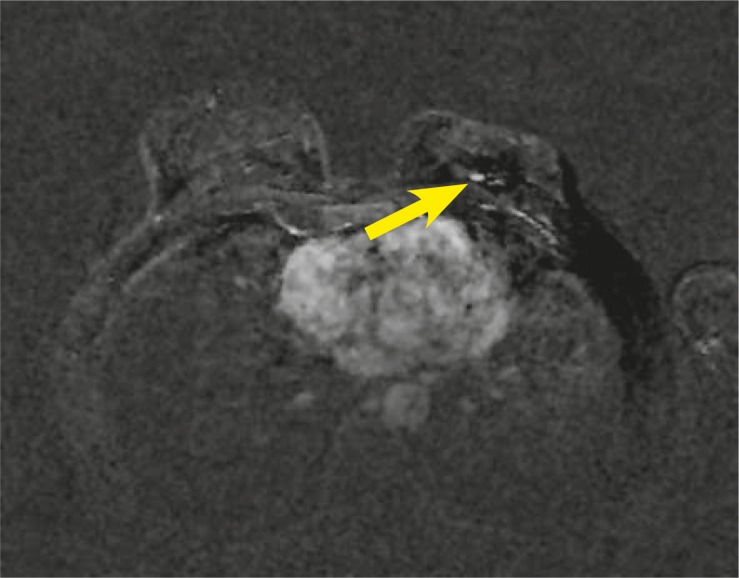
Axial T1-weighted image after Gadolinium injection (2^nd^ minute) and subtraction, focal enhancement 7 × 5 mm in the left breast in the prepectoral region (arrowhead).

After a gadolinium injection and subtraction a bilateral enhancement was seen: On the left side there was a 7 × 5 mm mass-like enhancement in the scar area. The margins were round and well circumscribed, the enhancement was homogeneous, and kinetic was 173% initial enhancement with plato BI-RADS 2 (Figure 2).

**FIGURE 3.**
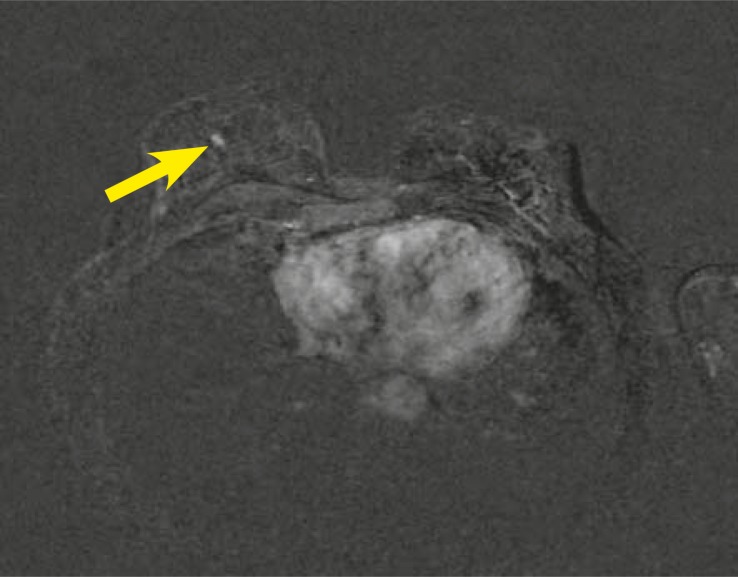
Axial T1-weighted image after Gadolinium injection (2^nd^ minute) and subtraction, ductal homogenous enhancement in the right breast 8 × 3 mm (arrowhead).

On the right there was a non-masslike enhancement. The enhancement pattern was ductal-linear in distribution measured 8 x 3mm. The internal enhancement was homogenous -BI-RADS 3–4 (Figure 3).

**FIGURE 4.**
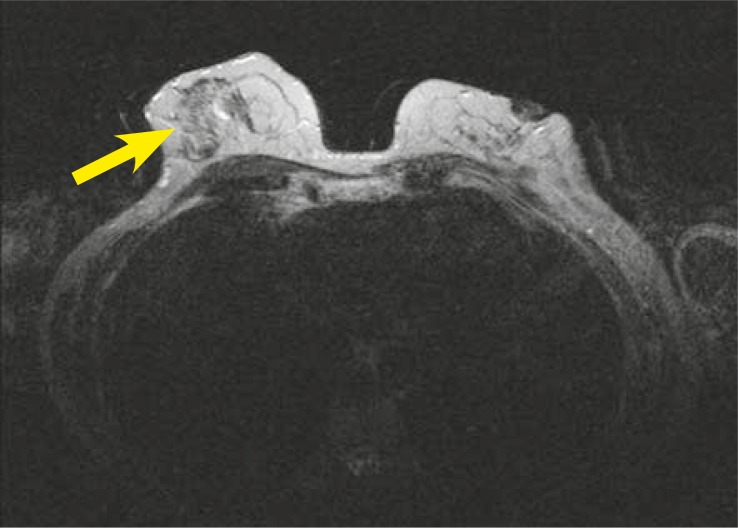
Axial T2-weighted image, a hiperintensive signal in the right breast (arrowhead).

On the precontrast T2-weighted sequence there was a hiperintensive signal in the area of ductal enhancement in the right breast. There were small cysts bilaterally (Figure 4).

**FIGURE 5.**
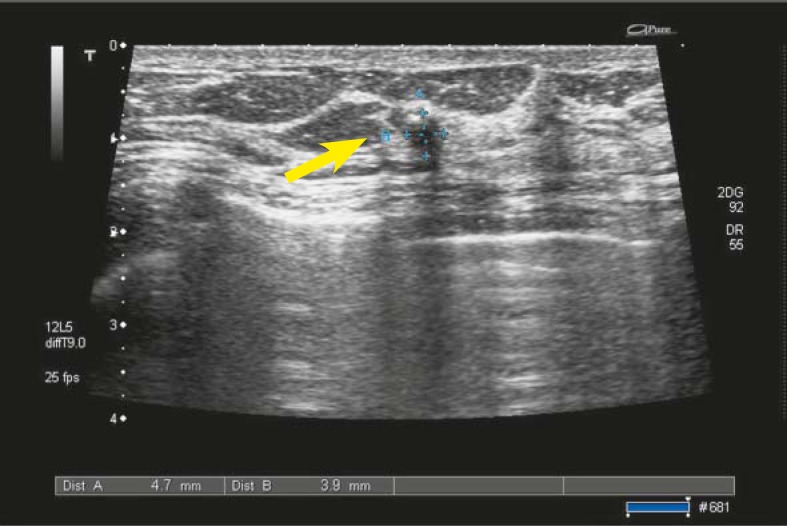
Small lesion in the left breast 5 × 4 mm, transonic with unsharp margins, vertically orientated, BI-RADS 4 (arrowhead).

The targeted ultrasound was performed, using 5–12 MHz linear transducer (Toshiba Aplio, Nasu, Japan). In the right breast there was no pathology. In the left breast there was a small tumour 5 × 4 mm categorized as BI-RADS 4 (Figure 5). The fine needle US guided biopsy was performed and cytology was inconclusive. During the procedure the patient was very anxious and difficult to communicate with.

Because of the MRI finding in the right breast (mammogaphicaly occult, targeted ultrasound negative) and because of the patient’s history the MRI-guided core biopsy was performed. MRI-guided vacuum-assisted breast biopsy was performed with MRI-supported Breast Immobilization and Biopsy System with the 4-channel breast coil in prone position. Axial T1-weighted images were acquired using a 3D FLASH through both breasts. Precontrast images were obtained in the axial plane with a slice thickness of 1.0 mm with distance factor 20%. Twenty seconds after contrast agent had been injected, another axial T1-3D FLASH sequence was performed with an injection of 0.1 mmol/kg of body weight of gadopentetate dimeglumine. Biopsy was performed with a 9-gauge MRI compatible vacuum-assisted biopsy. The biopsy site was marked with a titanium clip. “Postclip” axial 3D FLASH was performed to assess clip deployment.

The histological finding was DCIS-high grade, without any calcification. The patient was operated. The breast conserving therapy was performed. The clip in the right breast was localized by the radioguided occult lesion localization (ROLL) method under X-ray guidance. The lesion in her left breast was localized by ROLL method under US guidance. The pathologic results were the remnant foci of high-grade DCIS in the right breast and benign changes in the left breast.

## Discussion

The screening MRI has not yet been included in surveillance for patients treated by a breast-conserving therapy. However, the patient visited her doctor earlier because of changes in her right nipple, what demonstrated the importance of the breast-self examination.^6^ In addition, in our case MRI was performed because the patient had retracted nipple and dense breast.^3^ DCIS was represented as a ductal-linear homogenous enhancement on MRI images. The ductal-linear homogenous enhancement is a type of non-masslike enhancement.^7^,^8^ The path of enhancement follows the galactophoric system. The internal feature of the enhancement was homogenous in our case. DCIS and inflammatory disease are the most common causes for such a type of enhancement. The targeted ultrasound was negative, as we had expected.

Among the non-masslike enhancement detected initially on MRI, only 11% could be retrospectively detected by ultrasound and sonographically occult lesions have 22% probability of malignancy.^9^–^12^ Although the ductal enhancement was small, it measured only 8 × 3 mm, we decided to perform MRI-guided core biopsy and the histological result was conclusive.^13^,^14^ There was also a lesion which was incidentally found in the scar area of the left breast, which finally proved to be benign.

High-grade DCIS with no calcifications is not easy to diagnose by mammography due to the lack of typical malignant calcifications or masses, especially in dense breasts. Calcifications with or without mass are more common in women under 50 years.^11^ Autopsy studies have shown that almost 9% of women have undetected DCIS.^15^

Almost all invasive carcinomas are believed to begin as DCIS lesions but the time course of transition is unknown. Whether all DCIS will ultimately evolve to the invasive disease is unclear.^16^,^17^ In 2007 an article was published, that sensitivity of MRI for high-grade DCIS is much higher than that of mammography (92% vs. 56%), especially for high grade DCIS without necrosis (97% vs. 35%).^4^ If we pick up all cases of DCIS we would prevent virtually all cases of breast cancer, including CLB. CLB is the most common second primary breast cancer in patients diagnosed with breast cancer. The annual risk of developing any CLB remains constant at 0.75% per year after the treatment and persists for at least 20 years. The majority of patients (83%) harbouring CLB tumours develop invasive disease.^2^ The detection of second breast cancers in the asymptomatic phase leads to the detection of early-stage cancer and it improves the relative survival alike in other cancer’s localisations between 27% to 47%.^18^,^19^

In conclusion, by the Breast MRI Guidelines from the European Society of Breast Imaging^14^, currently there is not sufficient evidence to recommend the screening with MRI to patients treated by breast conserving surgery. But we might say that our case, in accordance to the European Guidelines, justifies MRI as a problem-solving modality when: the findings of conventional imaging are inconclusive and it is impossible to image sufficiently the primary tumour region after the conservative therapy with mammography.
